# Deep learning and radiomic feature-based blending ensemble classifier for malignancy risk prediction in cystic renal lesions

**DOI:** 10.1186/s13244-022-01349-7

**Published:** 2023-01-11

**Authors:** Quan-Hao He, Jia-Jun Feng, Fa-Jin Lv, Qing Jiang, Ming-Zhao Xiao

**Affiliations:** 1 Department of Urology, The First Affiliated Hospital of Chongqing Medical University, Chongqing, 400016 People’s Republic of China; 2 Department of Medical Imaging, Guangzhou First People’s Hospital, School of Medicine, South China University of Technology, Guangzhou, 51000 People’s Republic of China; 3 Department of Radiology, The First Affiliated Hospital of Chongqing Medical University, Chongqing, 400016 People’s Republic of China; 4 Department of Urology, The Second Affiliated Hospital of Chongqing Medical University, Chongqing, 400010 People’s Republic of China

**Keywords:** Machine learning, Bosniak-2019 classification, Cystic renal lesions, Radiomics

## Abstract

**Background:**

The rising prevalence of cystic renal lesions (CRLs) detected by computed tomography necessitates better identification of the malignant cystic renal neoplasms since a significant majority of CRLs are benign renal cysts. Using arterial phase CT scans combined with pathology diagnosis results, a fusion feature-based blending ensemble machine learning model was created to identify malignant renal neoplasms from cystic renal lesions (CRLs). Histopathology results were adopted as diagnosis standard. Pretrained 3D-ResNet50 network was selected for non-handcrafted features extraction and pyradiomics toolbox was selected for handcrafted features extraction. Tenfold cross validated least absolute shrinkage and selection operator regression methods were selected to identify the most discriminative candidate features in the development cohort. Feature’s reproducibility was evaluated by intra-class correlation coefficients and inter-class correlation coefficients. Pearson correlation coefficients for normal distribution and Spearman's rank correlation coefficients for non-normal distribution were utilized to remove redundant features. After that, a blending ensemble machine learning model were developed in training cohort. Area under the receiver operator characteristic curve (AUC), accuracy score (ACC), and decision curve analysis (DCA) were employed to evaluate the performance of the final model in testing cohort.

**Results:**

The fusion feature-based machine learning algorithm demonstrated excellent diagnostic performance in external validation dataset (AUC = 0.934, ACC = 0.905). Net benefits presented by DCA are higher than Bosniak-2019 version classification for stratifying patients with CRL to the appropriate surgery procedure.

**Conclusions:**

Fusion feature-based classifier accurately distinguished malignant and benign CRLs which outperformed the Bosniak-2019 version classification and illustrated improved clinical decision-making utility.

**Supplementary Information:**

The online version contains supplementary material available at 10.1186/s13244-022-01349-7.

## Key points

1. Blending ensemble model achieved excellent diagnostic performance in the external validation dataset.
2. Blending ensemble model exceeded the management performance based on the Bosniak classification.
3. Histopathology criterion support ensured that blending ensemble model’s predictions were reliable.

**Supplementary Information:**

The online version contains supplementary material available at 10.1186/s13244-022-01349-7.

## Background

The detection rate of cystic renal lesions (CRLs) is rising quickly as computed tomography (CT) becomes increasingly prevalent. A minority of CRLs are malignant renal neoplasms requiring surgical intervention. Cystic renal neoplasms present a broad category of kidney tumors with a wide range of biological profiles according to the WHO kidney tumor classification and the necessity of early surgical treatment for malignant CRL cannot be overstated [1]. However, the majority of CRLs are simple renal cysts or benign cystic renal neoplasms, which do not necessitate a radical surgery procedure like partial or radical nephrectomy. Since the components of CRL must be accurately identified in order to determine the appropriate treatment strategies, CT imaging is commonly utilized to differentiate CRL. Meanwhile, malignant CRL are difficult to diagnose and manage, especially in the early stage, due to their complex pattern on CT images including thickness of septation, enhancement of the mural nodule, calcifications, and etc. [2]. In an effort to identify malignant CRL at an early stage, standardize the terminology explaining complicated renal cysts, and offer criteria for classifying radical surgery-required malignant CRL, the Bosniak classification system was established [3, 4]. The updated 2019 version of the Bosniak classification system introduced more discriminative and quantitative criteria to improve the specificity in identifying higher risk CRL categories. In addition, it explicated detailed meanings of key terms to promote agreement and consistency among different readers. Based on the updated Bosniak-classification, one or more enhancing nodules in the CRL with obtuse margins (more than 4 mm) or with acute margins indicate a malignant renal neoplasm. Thickened wall or septa with enhancement in CRL also indicate the possibility of malignancy. However, these high-risk CRLs (IIF, III, IV) according to Bosniak classification could still be benign CRLs rather than malignant neoplasms. Inaccurate treatment and associated diagnostic errors caused by the misapplication of Bosniak categorization may lead to excessive medical care following adverse results like renal function impairment, re-operation surgery and medical disputes [5, 6]. It has been demonstrated that the diagnostic performance of 2019-Bosniak classification criteria do not significantly improve over its previous version [7–9]. According to the 2019-Bosniak version, a considerable proportion of previously diagnosed Class III lesions will be reclassified as IIF, resulting in lower sensitivity [10, 11]. Bosniak grades I and II are most commonly renal cysts, while grades IIF, III, and IV are more frequently malignant renal neoplasms. The latest study concluded that approximately 10%-20% of Bosniak IIF lesions, 50% of Bosniak III lesions, and 90% of Bosniak IV lesions were malignant renal neoplasms [12]. To improve diagnostic sensitivity and overcome the limitations of biased visual image evaluation, quantitative image analysis techniques, also known as radiomics, combined with machine learning methods have gained popularity in recent years [13, 14]. The purpose of this research is to develop and validate a blending ensemble machine learning algorithm for stratifying malignant and benign CRLs with the combination of deep learning and radiomic features.

## Materials and methods

### Enrollment criteria and characteristic distribution

**Fig. 1:**
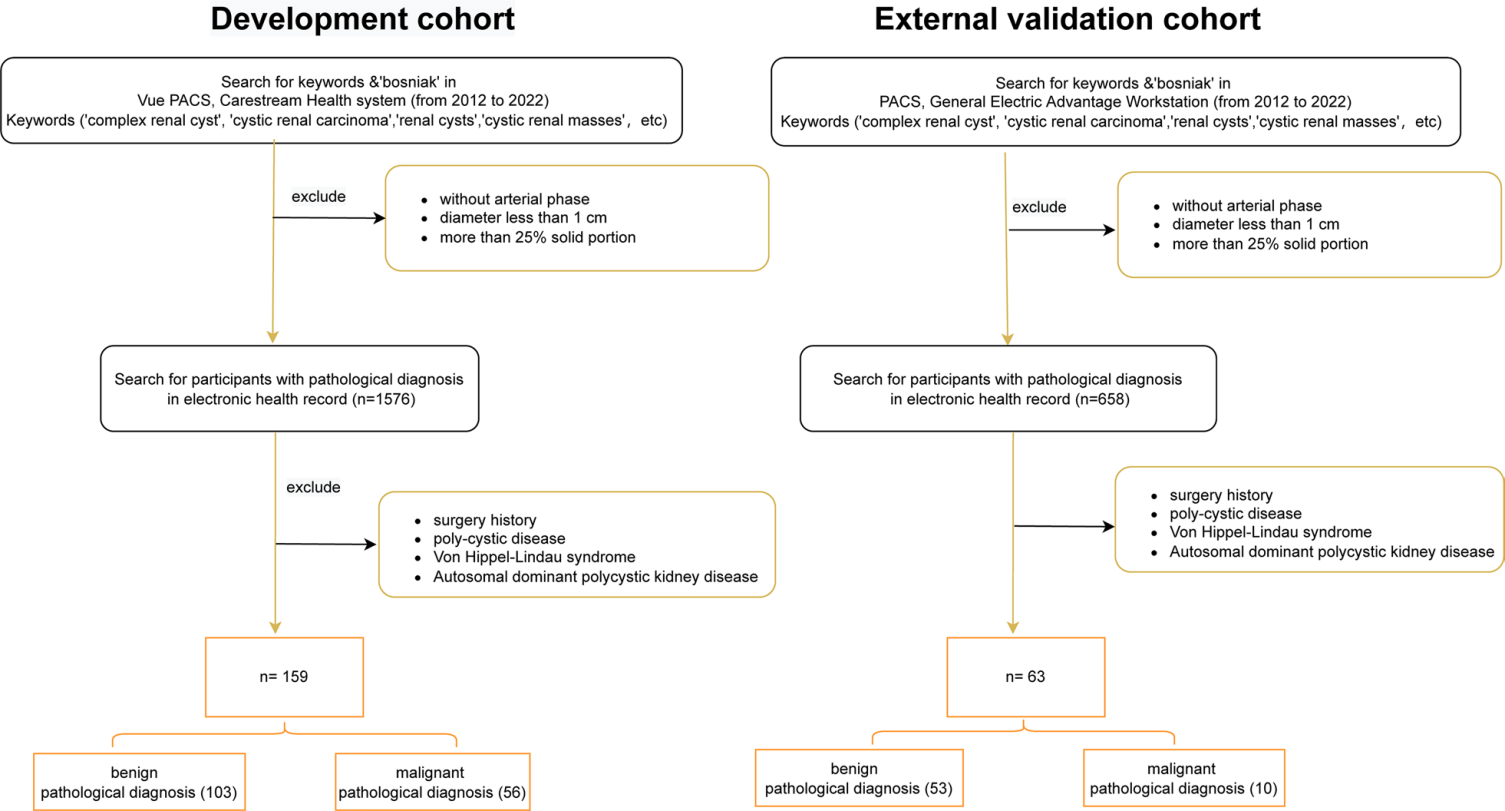
Flowchart representing how CRLs were enrolled and corresponding distribution of CRLs pathology results. Detailed inclusion and exclusion criteria are displayed in the flowchart. CRLs were classified as benign or malignant CRLs based on pathological results. Following that, training cohort were adopted to build machine learning classifier and testing cohort were used to evaluate model performance compared with Bosniak-2019 version

This retrospective analysis was approved by each hospital’s ethics committees, and all patient information was anonymized. In the training cohort, required CT scans were obtained from 128-slice spiral CT scanners (Siemens Healthcare, Germany) or 64-slice spiral CT scanners (General Electric, USA). In the testing cohort, required CT scans were obtained from a 128-slice spiral CT scanner (LightSpeed VCT, GE Medical Systems, USA). CT data were generated from the standardized scanning protocols. Details are as follows: CT-tube voltage (120–140 kv), CT-tube current (125–300 mAs), scanning matrix (512*512 pixels), body reconstruction kernel and slice thickness (ranging from 1 to 5 mm). After intravenous administration of iohexol (300 mg/mL at a rate of 3.0 mL/s, followed by a 30-mL saline flush), contrast-enhanced CT samples were captured. We retrieved CT images from the corresponding picture archiving and communication systems (Vue PACS, Carestream Health Inc & General Electric Advantage Workstation). Candidate participants included those with renal cysts exceeding 1 cm, without surgery history (renal needle biopsy, nephrolithotomy, nephrectomy or partial nephrectomy), without conditions associated with multiple renal cysts like poly-cystic disease, Von Hippel-Lindau syndrome (VHL) or Autosomal dominant polycystic kidney disease (ADPKD), and less than 25% solid portion in CRL. Each participant in this study could only include CRL confirmed by final pathology results, ensuring a realistic and reliable model’s presentation. Figure 1 depicts the detailed selection method and pathological results in two cohorts. 103 participants in the development cohort were diagnosed with benign CRL and 56 participants were diagnosed with malignant CRL. In the testing cohort, 10 participants were identified to have malignant CRL and 53 participants were identified to have benign CRL according to the pathological results. Table 1 shows detailed characteristic distributions in the training and testing cohort.

**Table 1:** Detailed distribution of Bosniak-2019 classification and pathology results in the training cohort and external validation cohort

Pathology analysis	Benign results	Malignance results
	*n* = 103	*n* = 56
*Training cohort*		
Bosniak I (*n* = 59)	Simple renal cysts (*n* = 59)	(*n* = 0)
Bosniak II (*n* = 23)	Simple renal cysts (*n* = 21)	Papillary renal cell carcinoma (*n* = 1) tubulocystic renal cell carcinoma (*n* = 1)
Bosniak	Simple renal cysts (*n* = 9)	Tubulocystic renal cell carcinoma (*n* = 2)
IIF	Cystic nephroma (*n* = 1)	Papillary renal cell carcinoma (*n* = 1) clear cell renal cell carcinoma (*n* = 3)
(*n* = 22)	Renal angiomyolipoma (*n* = 2)	Multilocular cystic renal neoplasm of low malignant potential (*n* = 4)
Bosniak	Simple renal cysts (*n* = 5)	clear cell renal cell carcinoma (*n* = 6) papillary renal cell carcinoma (*n* = 1)
III	Cystic nephroma (*n* = 1)	Chromophobe renal cell carcinoma (*n* = 1) tubulocystic renal cell carcinoma (*n* = 1)
(*n* = 18)	Renal angiomyolipoma (*n* = 1)	Multilocular cystic renal neoplasm of low malignant potential (*n* = 2)
Bosniak	Renal angiomyolipoma (*n* = 2)	Unclassified renal cell carcinoma (*n* = 5) clear cell renal cell carcinoma (*n* = 19)
IV		Papillary renal cell carcinoma (*n* = 5) chromophobe renal cell carcinoma (*n* = 2)
(*n* = 37)	Cystic nephroma (*n* = 2)	Multilocular cystic renal neoplasm of low malignant potential (*n* = 2)

	*n* = 53	*n* = 10
*Testing cohort*		
Bosniak I (*n* = 19)	Simple renal cysts (*n* = 19)	(*n* = 0)
Bosniak II (*n* = 11)	Simple renal cysts (*n* = 11)	(*n* = 0)
Bosniak IIF	Simple renal cysts (*n* = 16)	Clear cell renal cell carcinoma (2)
(*n* = 20)	Mixed epithelial and stromal tumor (1)	Multilocular cystic renal neoplasm of low malignant potential (1)
Bosniak III (*n* = 6)	Simple renal cysts (*n* = 5)	Clear cell renal cell carcinoma (1)
Bosniak IV (*n* = 7)	Cystic nephroma (1)	Clear cell renal cell carcinoma (5)
		Multilocular cystic renal neoplasm of low malignant potential (1)

### Handcrafted radiomic features extraction

All ROI labeling of CRLs was completed by two senior radiologists using ITK-SNAP software. When labeling the tumor margin, the radiologists will integrate image information in 3 different planes: the axial, sagittal, and coronal planes. In the case of contentious CRL sketching, another senior radiologist will participate in the discussion and help develop the final sketching results together. Radiomic features can be separated into three classes: (1) first-order statistics, (2) shape features, and (3) second-order features. Image types of radiomic Features can be classified into three categories: (1) Original, (2) Log, and (3) wavelet. Using the default parameters setting provided in the official Pyradiomics yaml file, we extracted 1231 radiomic features from each individual.

### Deep learning features extraction

**Fig. 2:**
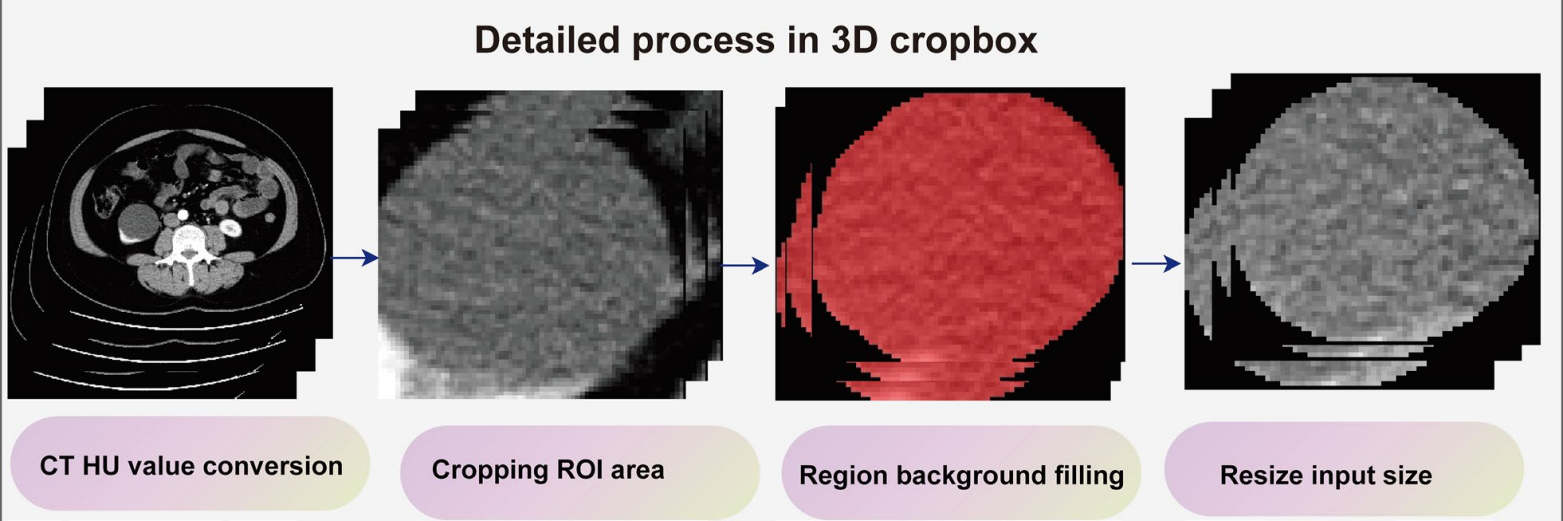
Detailed workflow of the 3D-cropbox. 3D-cropbox consists of four parts: CT HU conversion, ROI area cropping, region background filling, and input size tuning. The area outside the ROI will be filled with black to assure that deep learning features retrieved from the 3D-cropbox are entirely from CRL

For extracting deep learning features, we defined a 3D-cropbox to contain CRL area. The width and length of 3D-cropbox correspond to the maximum cross-sectional area of the CRL, while the height of 3D-cropbox corresponds to the dimensions containing the CRL region in the Z-axis. In the 3D-cropbox, NumPy array values outside ROI areas will be assigned to 0. Figure 2 displays the detailed 3D-cropbox workflow. The 3D-cropbox region will be transferred into a 3DResnet50 model with pre-trained weights. We extracted 2048 deep learning features from each individual by removing the last layer of the pre-trained model, disabling gradient updates and adding a 3D maximum pooling layer. The detailed 3DResnet50 structure is depicted in the Additional file 1: table2.

### Radiomic features harmonization

Genomic related research has widely adopted combat methods to deal with the batch effect. CT acquisition and reconstruction parameters have a direct impact on handcrafted radiomic features [15]. However, it is not realistic to standardize platforms and parameters in advance across different institutions. There is mounting evidence that radiomics research requires the same strategy [16, 17]. In this study, combat harmonization methods were adopted to address the difference in extracted radiomic features originated from different image acquisition procedures.

### Correlation coefficients test

To verify whether the selected features are highly reproducible and reliable, the intra-class correlation coefficients and inter-class correlation coefficients were employed. Results of the inter-class correlation coefficients were originated from two independent readers who re-labeled 25% participants CRLs in the training and testing cohorts. These re-labeled participants are randomly picked by an additional independent radiologist. Results of the intra-class correlation coefficients were estimated by one reader who randomly outlined same participants in the enrolled datasets at different times (1 month interval) [18].

### Quality control procedures

The quality control process for fusion features extraction and model construction consists of five steps: (1) Quality control of images; (2) quality control of ROI; (3) quality control of feature extraction; (4) quality control of feature selection; and (5) quality control of machine learning methods. We followed by the advice provided by the Image Biomarker Standardization Initiative (IBSI) [19]. Radiomics quality score (RQS) was adopted to assess the reliability in this research [20]. In the Additional file 1, detailed quality control procedures and RQS calculation results were presented.

### Statistical analysis

**Fig. 3:**
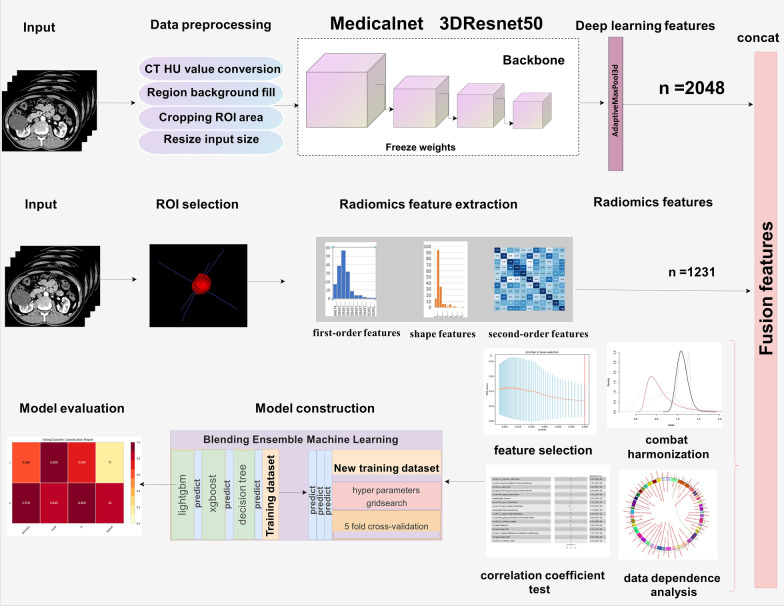
Flowchart presented the step-by-step procedures in machine learning model construction

ITK-SNAP (version 3.6.0) was used to generate ROI. Pyradiomics package (version 3.0.1) was used to extract handcrafted radiomic features. The pretrained weights in 3DResnet50 model are from 23 medical datasets (including brain MR images and lung CT images, etc.). The pretrained weights file and corresponding codes have been an open source published in Tencent Medicalnet project (https://github.com/Tencent/MedicalNet). Deep learning features were extracted by adding a 3D max-pooling layer and removing the upsampling layer in 3DResnet50 model. After feature extraction, the least absolute shrinkage and selection operator (LASSO) method with tenfold cross-validation was selected to choose the most identifiable features in the training datasets [21, 22]. Pearson correlation coefficients for normal distribution and Spearman's rank correlation coefficients for non-normal distribution were utilized to check for redundancy in the primary selected handcrafted radiomics features and deep learning features. Figure 3 depicts the entire procedure for model construction. The Receiver Operating Characteristics (ROC) curve and the accuracy score (ACC) are utilized to evaluate the final model’s performances. DeLong test is used to determine whether there was statistically considerable heterogeneity in the area under the receiver operating characteristic curve (AUC). Calibration curve is adopted to evaluate consistency performances of the final model in the external validation dataset. Decision curve analysis (DCA) is adopted to assess the clinical applicability compared with Bosniak-2019 version. The level of statistical significance is determined by two-sided p value of less than 0.05. The scikit-learn package and “Pycaret” package are adopted to create the final machine learning model. All model construction and plot drawing are developed in python environment (3.9 version) and R software (4.0.5 version).

## Results

### Blending ensemble classifier performances in CRL classification

**Fig. 4:**
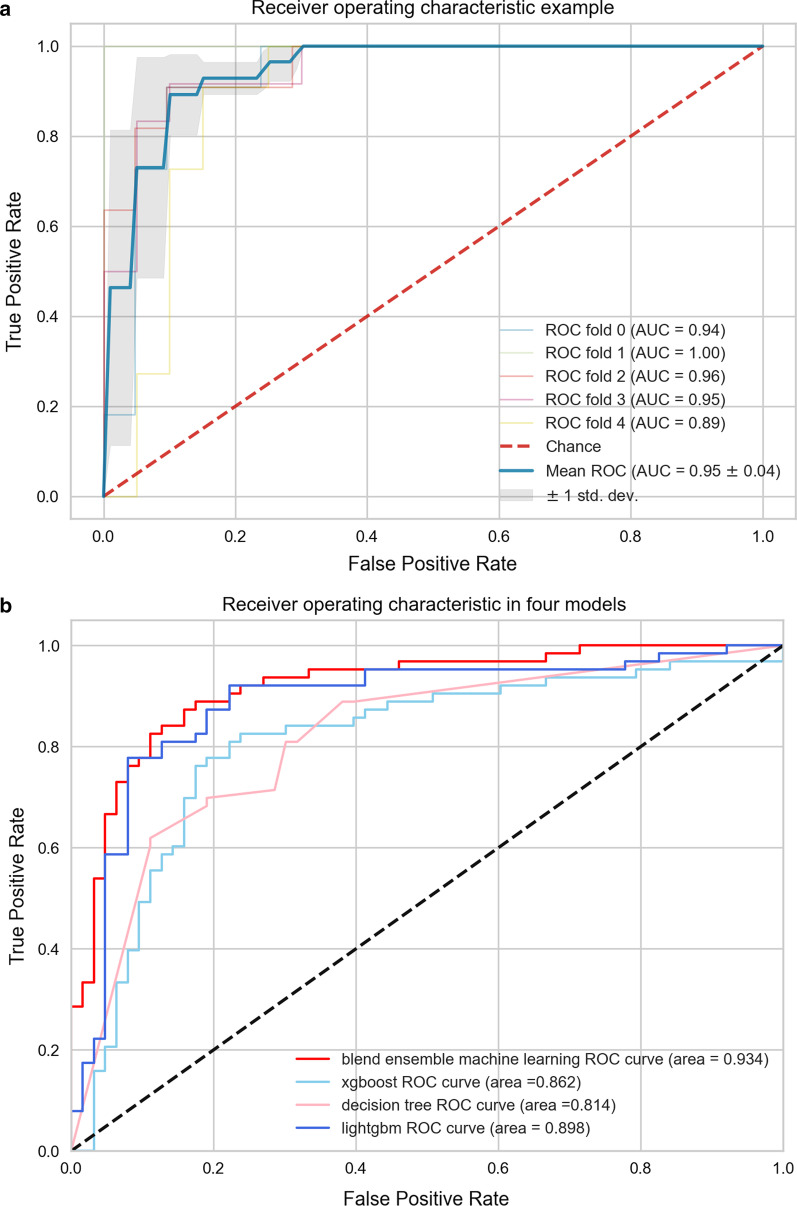
The diagnostic efficacy of each model assessed by ROC curve. **a** The mean cross-validated ROC of Blending ensemble model was 0.95. **b** All four models performed excellently in external validation dataset

**Fig. 5:**
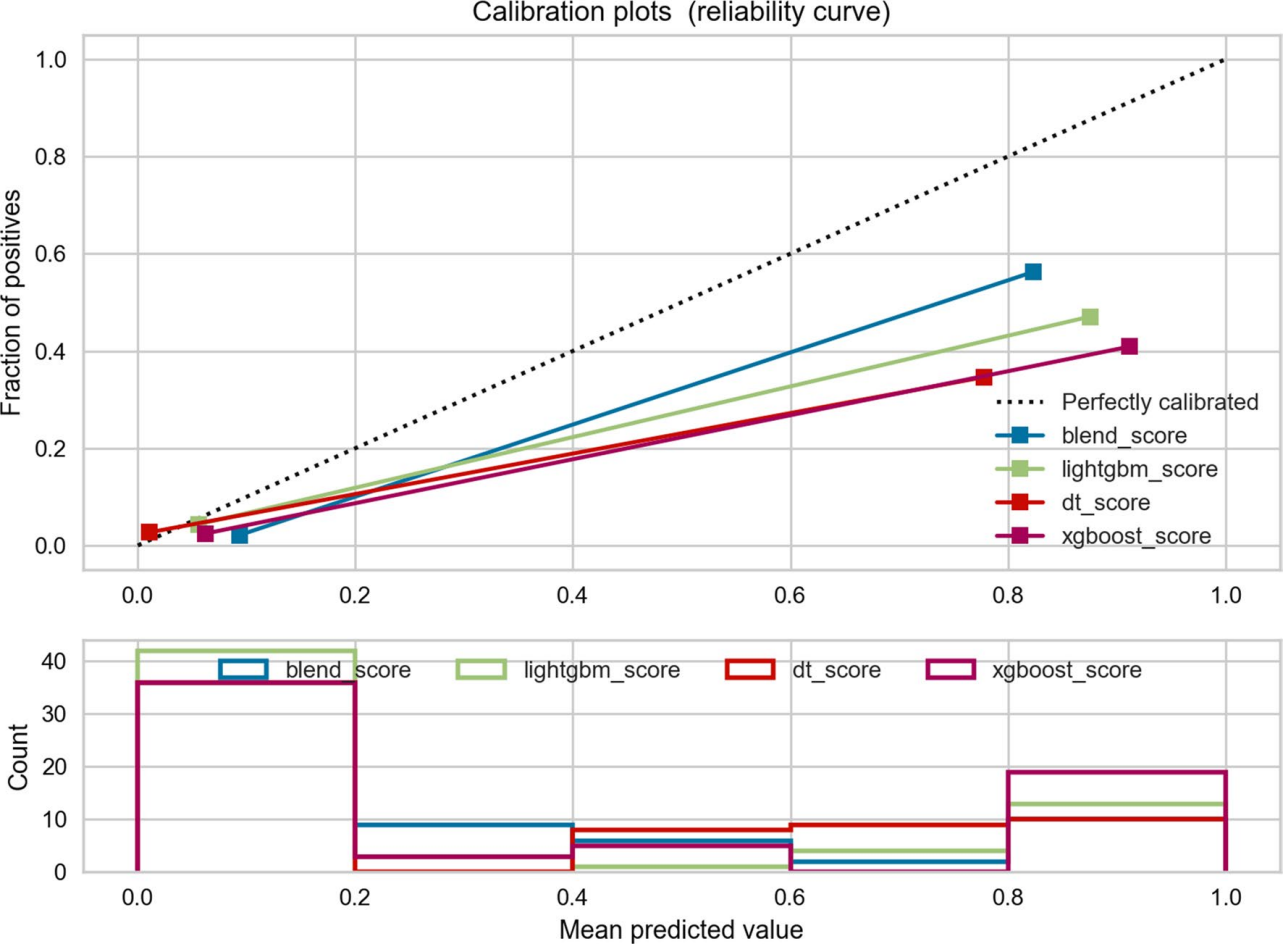
Calibration curve in external validation dataset. The black dashed line represents the ideal prediction curve. As the prediction curve of machine learning model approaches the dashed line, the model becomes more accurate. The average distributions of each probability in four models are displayed in the bar chart below

Detailed fusion-feature-based machine learning algorithm performances are displayed in Figs. 4 and 5, respectively. The AUC value in the final model is 0.934 which is statistically significant when compared to Bosniak classification according to the P value in the DeLong test (*p* < 0.001). ACC value in the ensemble model is 0.905 compared with Bosniak 2019 classification (ACC = 0.635), which demonstrated well discriminative ability in distinguishing malignant and benign CRLs. Detailed performances of the final model and Bosniak-2019-version classification are displayed in Table 2. Meanwhile, the fusion-feature-based machine learning algorithm displays strong calibration performance in Fig. 5.

**Table 2:** The performance of four models and Bosniak-2019 classification in external validation datasets

Model	Auc (95% CI)		Acc (95% CI)	Sensitivity	Specificity	*p* value in Delong test
*Train cohort fivefold cross-validation*						
Blending ensemble	0.946 (0.912–0.980)		0.899 (0.898–0.900)	0.893 (0.812–0.974)	0.903 (0.846–0.960)	*p* < 0.001
Decision tree	0.862 (0.800–0.924)		0.843 (0.841–0.844)	0.750 (0.637–0.863)	0.893 (0.834–0.953)	*p* = 0.770
lightgbm	0.950 (0.917–0.982)		0.893 (0.892–0.894)	0.946 (0.887–1.000)	0.864 (0.798–0.930)	*p* < 0.001
xgboost	0.938 (0.899–0.977)		0.906 (0.905–0.907)	0.893 (0.812–0.974)	0.913 (0.858–0.967)	*p* = 0.010
Bosniak 2019 classification	0.870 (0.823–0.918)		0.843 (0.841–0.844)	0.964 (0.916–1.000)	0.777 (0.696–0.857)	Reference
*Test cohort*						
Blending ensemble	0.934 (0.873–0.995)		0.905 (0.902–0.907)	0.900 (0.714–1.000)	0.906 (0.827–0.984)	*p* < 0.001
Decision tree	0.814 (0.681–0.947)		0.794 (0.789–0.799)	0.800 (0.552–1.000)	0.792 (0.683–0.902)	*p* = 0.681
lightgbm	0.898 (0.810–0.986)		0.905 (0.902–0.907)	0.800 (0.552–1.000)	0.925 (0.853–0.996)	*p* = 0.039
xgboost	0.862 (0.731–0.994)		0.841 (0.837–0.845)	0.900 (0.714–1.000)	0.830 (0.729–0.931)	*p* = 0.294
Bosniak 2019 classification	0.783 (0.716–0.850)		0.635 (0.628–0.642)	1.000 (1.000–1.000)	0.566 (0.433–0.699)	Reference

*Auc* area under the receiver operating characteristic curve, *Acc* accuracy score, *reference* reference in DeLong test, *95% CI* 95% confidence interval

### Clinical impact of blending ensemble classifier compared with Bosniak-2019 classification

**Fig. 6:**
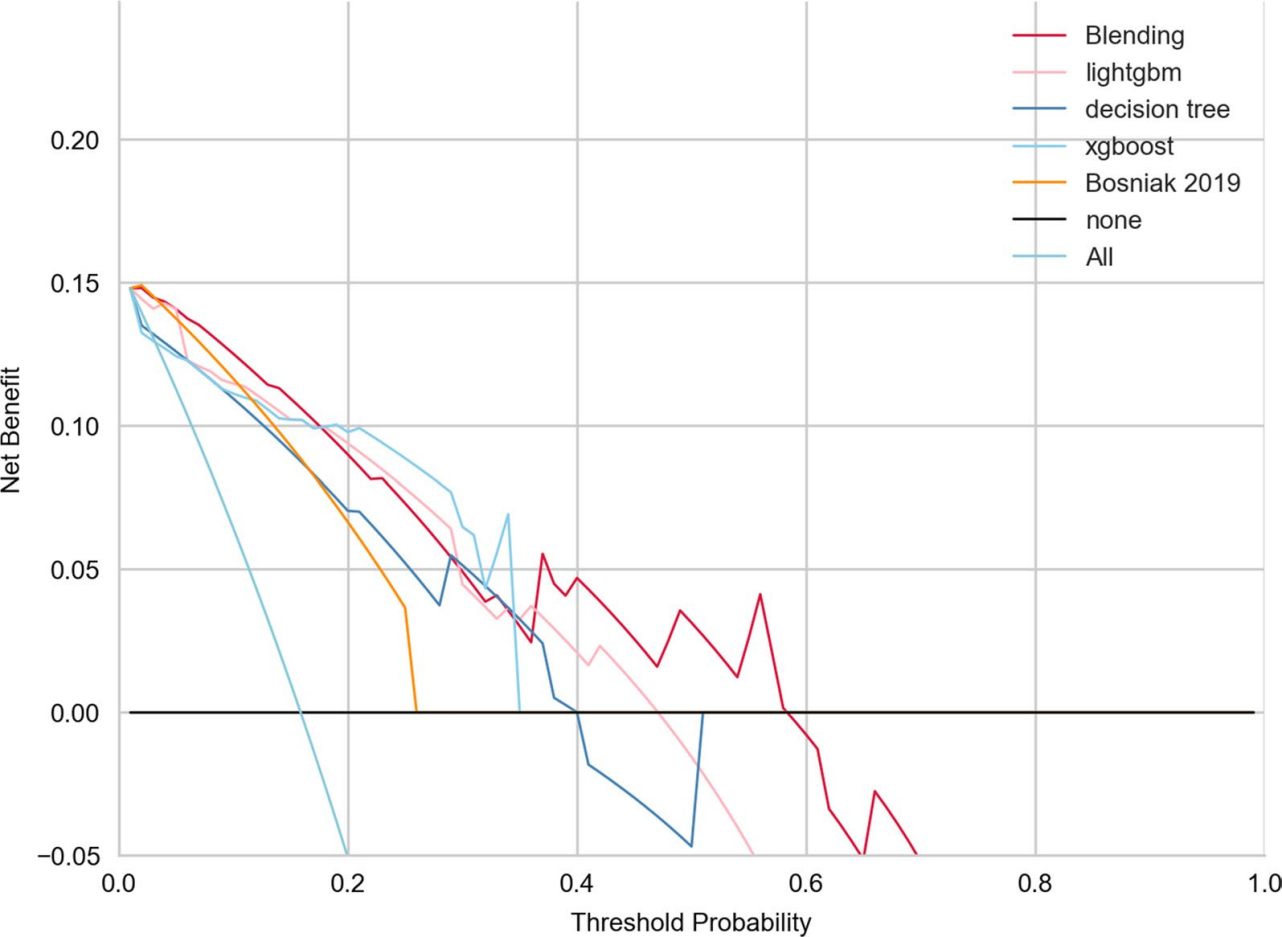
Decision curve analysis for four machine learning classifiers compared with Bosniak 2019 version in external validation dataset. The net benefit is represented on the y-axis and corresponding threshold probability is represented on the x-axis. The blending classifier is represented by the red line. The Bosniak 2019 version is represented by the yellow line. Compared with Bosniak 2019 version, all machine learning model performed better and gave more net benefits

**Fig. 7:**
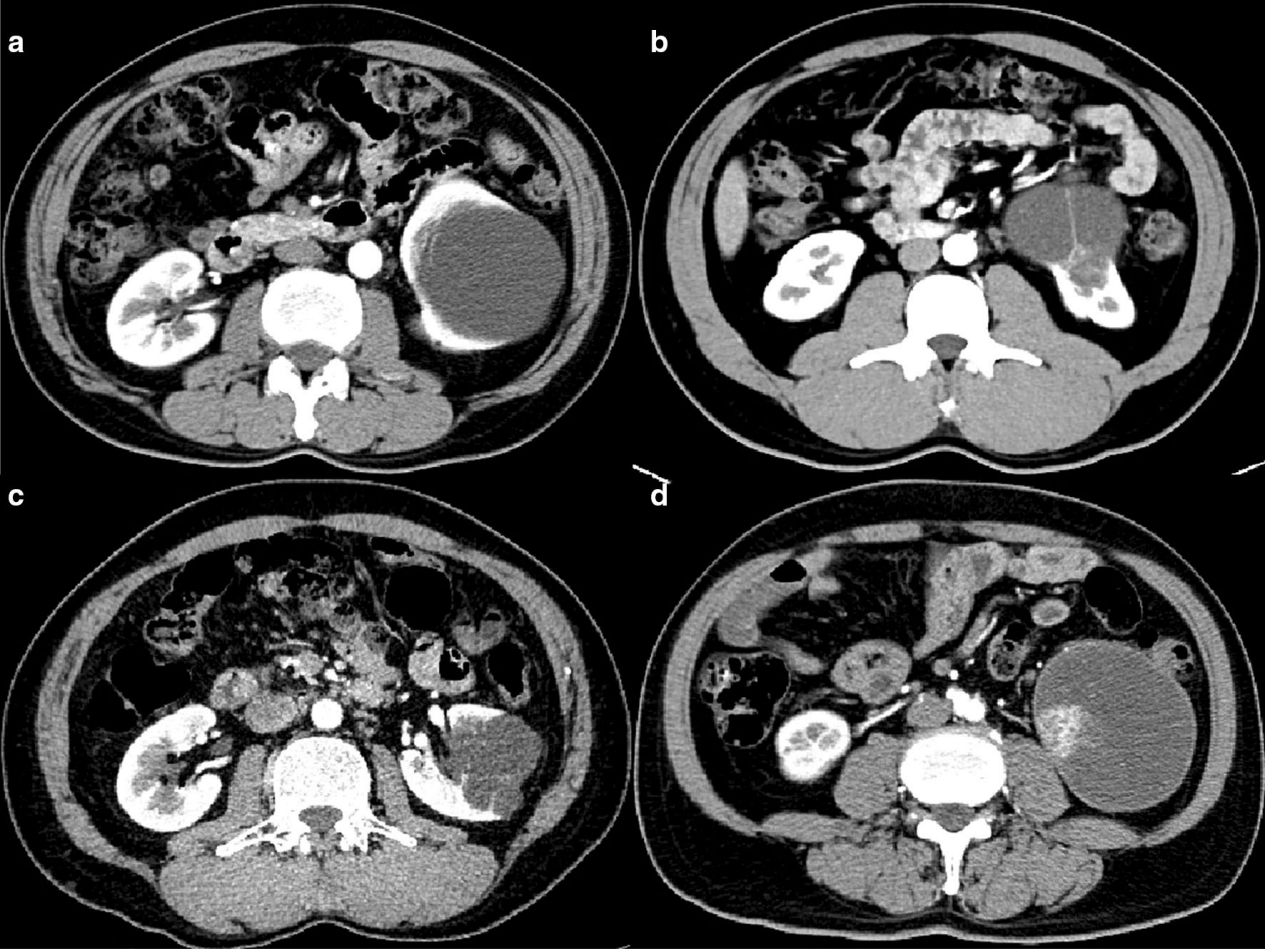
Arterial phase images for four cystic renal lesions in the external validation datasets. CRL in **a** is benign 2019 Bosniak II lesion and Bosniak IIF CRL in **b** is identified as multilocular cystic renal neoplasm of low malignant potential according to the histopathologic results after surgery. Cystic renal lesion in **c, d** were classified as 2019 Bosniak IIF CRL (benign pathology) and IV CRL (malignant pathology) respectively. Blending machine learning classifier all generated correct diagnosis

The decision curve analysis in external validation datasets for the final model find that, in any threshold probabilities, the fusion-features machine learning model will outperform "none" and "all" treatment strategies and deliver higher net benefit (Fig. 6). Figure 7 exemplifies the performance of the final model compared with Bosniak classification in testing dataset. The final model exceeds the management guideline based on the Bosniak 2019 classification in correctly classifying cystic renal lesions into malignant CRLs and benign CRLs in testing dataset. This suggests that using machine learning algorithm could provide better clinical decision support. Detailed confusion matrix for four models is displayed in the Additional file 1.

## Discussion

Although there is a strong association between the updated low-level Bosniak classification (Bosniak I, II) and benign CRL, it has limitations when evaluating the pathological results of Bosniak IIF, III, and IV labeled CRLs, which might result in unnecessary surgical procedures and excessive follow-up costs. Recognizing low malignant risk Bosniak classified high-level CRL can help avoid unnecessary treatment and increasing healthcare expenses [23, 24]. According to prior research, the progression of Bosniak IIF cystic renal masses is four years, which indicates a four years follow-up is inevitable [25]. Rapid progression of the high-risk Bosniak CRL necessitates radical nephrectomy rather than ineffective surgical procedures like renal cyst decortication [26, 27]. In this retrospective study, we employed a blending ensemble machine learning model to stratify malignant and benign CRLs in cystic renal masses, which outperformed the Bosniak classification system. We employed 3 deep learning features and 16 radiomic features in the final model which are reliable and discriminatory and performed robustly and consistently across internal validation and testing datasets. The reliability of blending ensemble model is determined by the following key elements: 1, IBSI guidelines are applied all across the design process. 2, Histopathologic examinations results are served as the diagnostic gold standard for CRL classification.3, A blending ensemble machine learning approach and cross-validation methods prevented overfitting in the training datasets.4, In the external validation step, the blending ensemble model demonstrated strong diagnostic performance 5. The RQS analysis result of this study is 16, which demonstrates that this study's quality is trustworthy and repeatable.

The updated 2019 version of the Bosniak classification intends to address inter-reader variability and improve diagnostic performance in predicting malignancy CRL. However, the proposed classification ability has yet to be confirmed [28]. Taking into account the pathologic reference standard, recent research indicates that Bosniak-2019 version IIF CRL have a higher malignancy risk than the previous Bosniak classification. Meanwhile, there are no variations in the proportion of malignancy when compared class III CRL with irregularities to class IV CRL with acute or obtuse nodules [29]. Nevertheless, there are still some disagreements between two well-trained radiologists in CRL Bosniak classification, which required an additional radiologist for help. As opposed to this, the blending decision algorithm performed well and consistently without the need for subjective evaluation across the testing dataset. Previous researches have demonstrated that machine learning approaches can be adopted for CRL malignancy stratification [30, 31]. Adopting first-order texture features (Mean, Entropy, Skewness and Kurtosis), Miskin et al. developed a radiomic-based machine learning method to classify cystic renal masses as benign cysts and potentially malignant cysts based on the Bosniak 2019 version reclassification [32]. However, they did not rely on pathology as the diagnostic criteria [33]. Bosniak classification is not as accurate as a pathological criterion and the Class IIF, III and IV CRLs could still be benign neoplasm, which means the model's clinical utility is constrained. Recently, Caroline Reinhold et al. employed a clinical decision algorithm to identify malignant renal neoplasms from CRLs [34]. CT-based machine learning model accurately stratified malignant CRL and outperformed Bosniak classification criterion. The decision-making system accurately distinguished CRL for active surveillance or required surgery and showed a net benefit across all threshold probabilities. However, the ability to distinguish benign and malignant CRLs remains debatable since benign CRLs were not defined by pathological standard while they defined benign CRL as non-imaging changes throughout a four-year follow-up. In order to ensure the reliability of the model performance, all enrolled CRLs in this study have post-operative pathological results. High specificity and sensibility have been demonstrated by blending algorithms, which could have an effect on clinical practice when radiologists or urologists try to assess and choose the best surgical approach for CRL.

Despite the fact that the final machine learning model successfully predicted the CRL pathology results, several restrictions should be mentioned. First, all CRLs ROI sketching were manually outlined by two radiologists and this approach looks like a bit out-fashioned. In recent researches, Kim et al. created a segmentation approach for measuring CRL, which is fully automated [35, 36]. In the follow-up study, to minimize the burden of radiologists and expand the applicability of the machine learning model, we will attempt to apply automated segmentation models like 3D-Unet or nn-Unet. Second, although external validation datasets were used in this work, the diagnostic performance of our machine learning model in large samples still has to be confirmed. Third, rather than employing Triple-phase CT scans, we adopted arterial phase CT images to build the machine learning method. Previous study adopted CNN and gated RNN model to distinguish malignant hepatic tumors based on multi-phase Contrast-enhanced computed tomography (CECT). The SpatialExtractor-TemporalEncoder-Integration-Classifier (STIC) successfully extracted the changing pattern across different CECT phases [37]. In Bosniak 2019 version, MRI standard criteria were formally introduced while there are very little researches focus on renal cysts textural features in MRI scans [38, 39]. Future researches could attempt to integrate the Triple-phase CT images and MRI images by sequence-to-sequence model like recurrent neural network (RNN) and vision transformer (VIT) [40]. In fact, cystic nephroma is more common in females aged 50–60 years, which indicates that clinical characteristic such as age and gender may be a possible predictor, and a mixture model that combines radiomics data with clinical features like STIC model may boost diagnostic model performance even further.

## Conclusion

In conclusion, a blending radiomics machine learning model demonstrated well discrimination capability in stratifying malignant and benign CRLs across testing datasets, which will benefit in diagnosing malignant CRL at an early stage and reducing overdiagnosis and overtreatment in CRL.

## Supplementary Information

**Additional file 1**. This material supplements detailed enrollment procedure and quality control methods.

## Data Availability

The datasets analyzed during the current study are not publicly available due to the need for follow-up research but are available from the corresponding author on reasonable request.

## Abbreviations

ACC: Accuracy score
AUC: The area under the receiver operating characteristic curve
CECT: Contrast-enhanced computed tomography
CRL: Cystic renal lesion
CT: Computed tomography
CTU: CT urography
ICCs: The intra-class correlation coefficients and inter-class correlation coefficients
IBSI: Image Biomarker Standardization Initiative’s suggestions
LASSO: Least absolute shrinkage and selection operator
RCC: Renal cell carcinoma
ROC: Receiver operating characteristic
ROI: Region of interests
RQS: Radiomics quality score
VIT: Vision transformer; RNN: recurrent neural network
