# Supplementary material for: Deep learning and radiomic feature-based blending ensemble classifier for malignancy risk prediction in cystic renal lesions
Source: Insights Imaging. 2023 Jan 11;14:6. doi: 10.1186/s13244-022-01349-7 (PMC9834471; doi:10.1186/s13244-022-01349-7)

ELECTRONIC SUPPLEMENTARY MATERIAL

"Deep learning and radiomic feature-based blending ensemble classifier for malignancy risk prediction in cystic renal lesions"

Detailed enrollment procedure and quality control methods.

Supplementary table1: The ICC value in the final selected 16 radiomic features and 3deep learning features.
Supplementary table2: Detailed structure components and parameters in 3Dresnet50 model
Supplementary figure1: The prior distributions of scale parameters (Delta) before and after handcrafted radiomic features harmonization
Supplementary figure2: The prior distributions of scale parameters (Gamma) before and after handcrafted radiomic features harmonization
Supplementary figure3: The error bar plot in lasso selection
Supplementary figure4 selected features weights after lasso selection
Supplementary figure5: Roc curves for blending voting classifier in external validation dataset.
Supplementary figure6: The confusion matrix for the blending model and base models in external validation datasets
Supplementary figure7: The circle plot shows the correlation between deep learning and handcrafted radiomic features after lasso selection.
Supplementary figure8: Decision boundary plot for four classifiers in external validation dataset.

Detailed enrollment procedure and quality control methods

Candidate participants enrollment procedure

In this study, all CT scans data in development cohort were originated from picture archiving and communication systems (PACS: RISGC version3.1s19.5, Carestream health Inc.). All CT scans data in external validation cohort were derived from General Electric Advantage Workstation. Corresponding pathology results for CRL were retrieved from each hospital's electronic pathology system or computer-based patient record (EMR). Keywords linked with CRL (cystic renal masses, cystic renal carcinoma, complex renal cysts, Cystic renal tumor, Bosniak, etc.) were used to initially select candidate CT scans in PACS system. The exclusion criteria included images without arterial phase, diameter of CRL less than 1cm, more than 25% solid portions. After that, based on the patient data from the PACS system, we searched the pathology results from the EMR and electric pathology system. The exclusion criteria included participants with renal surgery history and chronic conditions like poly-cystic disease, Von Hippel-Lindau syndrome (VHL) or Autosomal dominant polycystic kidney disease (ADPKD).

CRLs Bosniak-2019 version reclassification

Two professional abdominal radiologists independently reclassify CRLs in the training and external validation datasets according to the Bosniak-2019 version. They are blind to the corresponding pathological results. In the case of contentious CRLs classification, another senior radiologist will participate in the discussion and help develop the final decision together.

quality control procedures

CT scans quality control

In training cohort, contrast-enhanced CT scans were obtained from 128-slice spiral CT scanners (Siemens Healthcare, Germany) or 64-slice spiral CT scanners (General Electric, USA). In external validation cohort, enrolled patients underwent contrast-enhanced CT scans with a 128-slice scanner (LightSpeed VCT, GE Medical systems, USA). The standardized protocols in CT image scanning were as follows: Each patient was given to a three-phase scan (plain scan, arterial phase, and venous phase). CT scanning parameters including CT-tube voltage (120-140 kv), CT-tube current (125-300 mAs), scanning matrix (512*512 pixels), body reconstruction kernel, and slice thickness (ranging from 1mm to 5mm). After intravenous administration of iohexol (300 mg/mL at a rate of 3.0 mL/s, followed by a 30-mL saline flush), contrast-enhanced CT images were captured. The total contrast volume for each kilogram

was 1.5 ml. The arterial phases images were selected for further research.

ROI sketching quality control

Considering that manual ROI delineation is vulnerable to wide variation between observers, which may lead to unreliable results, we adhere to the ISBI recommendations for validating the repeatability of ROI regions. Feature reproducibility is evaluated by intra-class correlation coefficients and inter-class correlation coefficients (ICC), which are generated from radiomic features extracted by two independent ROI sketchers. ICC values >0.75 were considered as robust features in previous studies(1,2). In this study, ICC threshold was set to 0.75 and all 19 features in the training datasets were normalized prior to feature selection according to their mean value and standard deviation value (z-score normalization) to make sure the comparability of each selected variable (**Supplementary table1)**.

Feature extraction quality control

preprocessing for 3Dresnet 50 features extraction

Within the cropped area, the area outside the ROI will be fulfilled with black. After segmentation of the tumor region delineated, the informative slices (the consecutive axial slices containing full tumor area) are cropped and resized to 14 *128 * 128 dimensional NumPy format files (the size for the input layer of the 3Dresnet models). The cropped images will be selected as the input of convolutional neural network (CNN) model.

3Dresnet 50 network structure

Tencent Medicalnet's 3Dresnet model, which was pretrained on 23 medical datasets, is employed in this study to extract deep learning features. This model is publicly assessable as open-source code (https://github.com/Tencent/MedicalNet). After data pre-processing for deep learning features and model modification, the cropped CT scans were propagated in the network to generate deep learning features.

Handcrafted radiomic features parameters settings

Using the python Pyradiomics package, handcrafted radiomic features were created by manually sketched CRL ROI. Detailed calculation methods of handcrafted radiomic features are described and provided in online documentation of Pyradiomics (https://pyradiomics.readthedocs.io/en/latest/features.html). We start features extraction by using the standard sample parameters setting provided in the official Pyradiomics YAML file and all the images will be resampled to 1×1×1 mm^3 voxels to standardize the slice thickness.(3). Image intensities will be binned by 25 HU and voxel array shift is set to 1000. All radiomic features included in this investigation adhere to the Imaging Biomarker Standardization Initiative's feature criteria (IBSI).

Handcrafted radiomic features harmonization

Combat approaches are used to mitigate the multicenter impact caused by different CT scanner and protocol settings. To lessen the multicenter impact caused by varied CT scanner and protocol settings, combat methods are deployed. The nonparametric form model is adopted in Combat methods to determine the transformation for each feature separately by using “sva” R package (https://bioconductor.org/packages/release/bioc/html/sva.html). **Supplementary figure1 and figure2** depict the prior distributions of scale parameters (Gamma and Delta) before and after handcrafted radiomic features harmonization (4).

Candidate features selection quality control

All regions of interest (ROI) were generated from ITK-SNAP (version 3.6.0) and handcrafted radiomic features extraction was conducted using Pyradiomics package (version 3.0.1) in python environment (version 3.7)(5). 1231 radiomic features and 2048 deep learning features were generated in each individual at the beginning. In the development cohort, the least absolute shrinkage and selection operator (LASSO) which could add the penalty for non-zero coefficients to the sum of the absolute value (L1 penalty) were selected to filter the candidate variables. All candidate variables were normalized before LASSO selection. At the selected λ value of 0.0154, 46 candidate features were selected by LASSO methods (**supplementary figure3**). To eliminate redundancy in the primary selected candidate variables, the Spearman's correlation coefficient for variables with non-normal distribution and the Pearson correlation coefficient for variables with normal distribution were separately employed.

Machine learning algorithms quality control

Detailed radiomics quality score (RQS) calculation results

The radiomics quality score (RQS1.0 version) of this study reached 16(6). The cumulated points are obtained by complying with image protocol quality (+1), feature reduction or adjustment for multiple testing (+3), discrimination method with resampling method (+2), calibration statistics method (+1), validation from another institute (+3), comparison to “gold standard” (+2), potential clinical utility (+2), open-sourced code (+1), and open-sourced radiomic features (+1).

Correctness of each model and the corresponding confusion matrix

All four models showed well performance and satisfactory accuracy score (decision tree ACC=79.4%, lightgbm ACC=90.5%, Xgboost ACC=84.1%, blending algorithm ACC =90.5%). **Supplementary figure6** shows the corresponding confusion matrix for the blending model and base models in external validation datasets.

References

1. Graumann, O., Osther, S. S., Karstoft, J., Hørlyck, A., and Osther, P. J. (2015) Bosniak classification system: inter-observer and intra-observer agreement among experienced uroradiologists. *Acta radiologica (Stockholm, Sweden : 1987)* **56**, 374-383
2. Bartko, J. (1966) The Intraclass Correlation Coefficient as a Measure of Reliability. *Psycholog Rep* **19**
3. van Griethuysen, J. J. M., Fedorov, A., Parmar, C., Hosny, A., Aucoin, N., Narayan, V., Beets-Tan, R. G. H., Fillion-Robin, J.-C., Pieper, S., and Aerts, H. J. W. L. (2017) Computational Radiomics System to Decode the Radiographic Phenotype. *Cancer Research* **77**, e104-e107
4. Leek, J. T., Johnson, W. E., Parker, H. S., Jaffe, A. E., and Storey, J. D. (2012) The sva package for removing batch effects and other unwanted variation in high-throughput experiments. *Bioinformatics (Oxford, England)* **28**, 882-883
5. Yushkevich, P. A., Pashchinskiy, A., Oguz, I., Mohan, S., Schmitt, J. E., Stein, J. M., Zukić, D., Vicory, J., McCormick, M., Yushkevich, N., Schwartz, N., Gao, Y., and Gerig, G. (2019) User-Guided Segmentation of Multi-modality Medical Imaging Datasets with ITK-SNAP. *Neuroinformatics* **17**, 83-102
6. Lambin, P., Leijenaar, R. T. H., Deist, T. M., Peerlings, J., de Jong, E. E. C., van Timmeren, J., Sanduleanu, S., Larue, R. T. H. M., Even, A. J. G., Jochems, A., van Wijk, Y., Woodruff, H., van Soest, J., Lustberg, T., Roelofs, E., van Elmpt, W., Dekker, A., Mottaghy, F. M., Wildberger, J. E., and Walsh, S. (2017) Radiomics: the bridge between medical imaging and personalized medicine. *Nature Reviews Clinical Oncology* **14**, 749-762

Supplementary table1: The ICC values in the final selected 16 radiomic features and 3deep learning features.

feature label	ICC (interclass)	ICC (intraclass)
wavelet.LLL_glcm_Imc2	0.930(0.828-0.968)	0.994(0.989-0.997)
wavelet.LLL_firstorder_10Percentile	0.999(0.997-0.999)	0.989(0.979-0.994)
wavelet.LLL_glszm_SmallAreaEmphasis	0.979(0.942-0.991)	0.992(0.984-0.996)
wavelet.HHH_glszm_ZoneVariance	0.997(0.995-0.999)	0.999(0.998-1.000)
wavelet.HLH_glcm_ClusterShade	0.999(0.998-0.999)	0.998(0.997-0.999)
wavelet.LHH_glszm_SmallAreaEmphasis	0.929(0.870-0.962)	0.928(0.870-0.961)
wavelet.LLH_glrlm_LongRunLowGrayLevelEmphasis	0.997(0.995-0.999)	1.000(1.000-1.000)
original_shape_Flatness	0.992(0.985-0.996)	0.993(0.986-0.996)
log.sigma.5.0.mm.3D_glszm_GrayLevelNonUniformity	0.989(0.979-0.994)	0.994(0.988-0.997)
X3dresnet.feature785	0.956(0.919-0.977)	0.982(0.967-0.991)
wavelet.HHH_glszm_SizeZoneNonUniformityNormalized	0.978(0.958-0.988)	0.963(0.931-0.980)
X3dresnet.feature621	0.750(0.575-0.859)	0.965(0.935-0.981)
X3dresnet.feature1929	0.999(0.999-1.000)	0.997(0.994-0.998)
wavelet.LLL_gldm_SmallDependenceHighGrayLevelEmphasis	0.993(0.977-0.997)	0.999(0.998-0.999)
wavelet.LHL_firstorder_Mean	0.952(0.912-0.975)	0.993(0.987-0.996)
wavelet.LHL_firstorder_Median	0.974(0.952-0.986)	0.987(0.977-0.993)
wavelet.LLH_glrlm_RunEntropy	0.997(0.993-0.998)	1.000(1.000-1.000)
wavelet.LHH_gldm_DependenceEntropy	1.000(0.999-1.000)	1.000(1.000-1.000)
log.sigma.3.0.mm.3D_glcm_Imc1	0.991(0.982-0.995)	0.985(0.972-0.992)

The intraclass Correlation Coefficients and interclass correlation coefficients in the final selected 16 radiomic features and 3deep learning features. Inclusion criteria in this study is ICCs values greater than 0.75, CI: Confidence interval.

Supplementary table2: Detailed structure components and parameters in 3Dresnet50 model

3DResnet model structure					
**Layer (type)**	**Output shape**	**Parameters**	**Layer (type)**	**Output Shape**	**Parameters**
Conv3d-1	[-1, 64, 7, 64, 64]	21,952	BatchNorm3d-88	[-1, 1024, 2, 16, 16]	2,048
BatchNorm3d-2	[-1, 64, 7, 64, 64]	128	ReLU-89	[-1, 1024, 2, 16, 16]	0
ReLU-3	[-1, 64, 7, 64, 64]	0	Bottleneck-90	[-1, 1024, 2, 16, 16]	0
MaxPool3d-4	[-1, 64, 4, 32, 32]	0	Conv3d-91	[-1, 256, 2, 16, 16]	262,144
Conv3d-5	[-1, 64, 4, 32, 32]	4,096	BatchNorm3d-92	[-1, 256, 2, 16, 16]	512
BatchNorm3d-6	[-1, 64, 4, 32, 32]	128	ReLU-93	[-1, 256, 2, 16, 16]	0
ReLU-7	[-1, 64, 4, 32, 32]	0	Conv3d-94	[-1, 256, 2, 16, 16]	1,769,472
Conv3d-8	[-1, 64, 4, 32, 32]	110,592	BatchNorm3d-95	[-1, 256, 2, 16, 16]	512
BatchNorm3d-9	[-1, 64, 4, 32, 32]	128	ReLU-96	[-1, 256, 2, 16, 16]	0
ReLU-10	[-1, 64, 4, 32, 32]	0	Conv3d-97	[-1, 1024, 2, 16, 16]	262,144
Conv3d-11	[-1, 256, 4, 32, 32]	16,384	BatchNorm3d-98	[-1, 1024, 2, 16, 16]	2,048
BatchNorm3d-12	[-1, 256, 4, 32, 32]	512	ReLU-99	[-1, 1024, 2, 16, 16]	0
Conv3d-13	[-1, 256, 4, 32, 32]	16,384	Bottleneck-100	[-1, 1024, 2, 16, 16]	0
BatchNorm3d-14	[-1, 256, 4, 32, 32]	512	Conv3d-101	[-1, 256, 2, 16, 16]	262,144
ReLU-15	[-1, 256, 4, 32, 32]	0	BatchNorm3d-102	[-1, 256, 2, 16, 16]	512
Bottleneck-16	[-1, 256, 4, 32, 32]	0	ReLU-103	[-1, 256, 2, 16, 16]	0
Conv3d-17	[-1, 64, 4, 32, 32]	16,384	Conv3d-104	[-1, 256, 2, 16, 16]	1,769,472
BatchNorm3d-18	[-1, 64, 4, 32, 32]	128	BatchNorm3d-105	[-1, 256, 2, 16, 16]	512

ReLU-19	[-1, 64, 4, 32, 32]	0	ReLU-106	[-1, 256, 2, 16, 16]	0
Conv3d-20	[-1, 64, 4, 32, 32]	110,592	Conv3d-107	[-1, 1024, 2, 16, 16]	262,144
BatchNorm3d-21	[-1, 64, 4, 32, 32]	128	BatchNorm3d-108	[-1, 1024, 2, 16, 16]	2,048
ReLU-22	[-1, 64, 4, 32, 32]	0	ReLU-109	[-1, 1024, 2, 16, 16]	0
Conv3d-23	[-1, 256, 4, 32, 32]	16,384	Bottleneck-110	[-1, 1024, 2, 16, 16]	0
BatchNorm3d-24	[-1, 256, 4, 32, 32]	512	Conv3d-111	[-1, 256, 2, 16, 16]	262,144
ReLU-25	[-1, 256, 4, 32, 32]	0	BatchNorm3d-112	[-1, 256, 2, 16, 16]	512
Bottleneck-26	[-1, 256, 4, 32, 32]	0	ReLU-113	[-1, 256, 2, 16, 16]	0
Conv3d-27	[-1, 64, 4, 32, 32]	16,384	Conv3d-114	[-1, 256, 2, 16, 16]	1,769,472
BatchNorm3d-28	[-1, 64, 4, 32, 32]	128	BatchNorm3d-115	[-1, 256, 2, 16, 16]	512
ReLU-29	[-1, 64, 4, 32, 32]	0	ReLU-116	[-1, 256, 2, 16, 16]	0
Conv3d-30	[-1, 64, 4, 32, 32]	110,592	Conv3d-117	[-1, 1024, 2, 16, 16]	262,144
BatchNorm3d-31	[-1, 64, 4, 32, 32]	128	BatchNorm3d-118	[-1, 1024, 2, 16, 16]	2,048
ReLU-32	[-1, 64, 4, 32, 32]	0	ReLU-119	[-1, 1024, 2, 16, 16]	0
Conv3d-33	[-1, 256, 4, 32, 32]	16,384	Bottleneck-120	[-1, 1024, 2, 16, 16]	0
BatchNorm3d-34	[-1, 256, 4, 32, 32]	512	Conv3d-121	[-1, 256, 2, 16, 16]	262,144
ReLU-35	[-1, 256, 4, 32, 32]	0	BatchNorm3d-122	[-1, 256, 2, 16, 16]	512
Bottleneck-36	[-1, 256, 4, 32, 32]	0	ReLU-123	[-1, 256, 2, 16, 16]	0
Conv3d-37	[-1, 128, 4, 32, 32]	32,768	Conv3d-124	[-1, 256, 2, 16, 16]	1,769,472
BatchNorm3d-38	[-1, 128, 4, 32, 32]	256	BatchNorm3d-125	[-1, 256, 2, 16, 16]	512

ReLU-39	[-1, 128, 4, 32, 32]	0	ReLU-126	[-1, 256, 2, 16, 16]	0
Conv3d-40	[-1, 128, 2, 16, 16]	442,368	Conv3d-127	[-1, 1024, 2, 16, 16]	262,144
BatchNorm3d-41	[-1, 128, 2, 16, 16]	256	BatchNorm3d-128	[-1, 1024, 2, 16, 16]	2,048
ReLU-42	[-1, 128, 2, 16, 16]	0	ReLU-129	[-1, 1024, 2, 16, 16]	0
Conv3d-43	[-1, 512, 2, 16, 16]	65,536	Bottleneck-130	[-1, 1024, 2, 16, 16]	0
BatchNorm3d-44	[-1, 512, 2, 16, 16]	1,024	Conv3d-131	[-1, 256, 2, 16, 16]	262,144
Conv3d-45	[-1, 512, 2, 16, 16]	131,072	BatchNorm3d-132	[-1, 256, 2, 16, 16]	512
BatchNorm3d-46	[-1, 512, 2, 16, 16]	1,024	ReLU-133	[-1, 256, 2, 16, 16]	0
ReLU-47	[-1, 512, 2, 16, 16]	0	Conv3d-134	[-1, 256, 2, 16, 16]	1,769,472
Bottleneck-48	[-1, 512, 2, 16, 16]	0	BatchNorm3d-135	[-1, 256, 2, 16, 16]	512
Conv3d-49	[-1, 128, 2, 16, 16]	65,536	ReLU-136	[-1, 256, 2, 16, 16]	0
BatchNorm3d-50	[-1, 128, 2, 16, 16]	256	Conv3d-137	[-1, 1024, 2, 16, 16]	262,144
ReLU-51	[-1, 128, 2, 16, 16]	0	BatchNorm3d-138	[-1, 1024, 2, 16, 16]	2,048
Conv3d-52	[-1, 128, 2, 16, 16]	442,368	ReLU-139	[-1, 1024, 2, 16, 16]	0
BatchNorm3d-53	[-1, 128, 2, 16, 16]	256	Bottleneck-140	[-1, 1024, 2, 16, 16]	0
ReLU-54	[-1, 128, 2, 16, 16]	0	Conv3d-141	[-1, 512, 2, 16, 16]	524,288
Conv3d-55	[-1, 512, 2, 16, 16]	65,536	BatchNorm3d-142	[-1, 512, 2, 16, 16]	1,024
BatchNorm3d-56	[-1, 512, 2, 16, 16]	1,024	ReLU-143	[-1, 512, 2, 16, 16]	0
ReLU-57	[-1, 512, 2, 16, 16]	0	Conv3d-144	[-1, 512, 2, 16, 16]	7,077,888
Bottleneck-58	[-1, 512, 2, 16, 16]	0	BatchNorm3d-145	[-1, 512, 2, 16, 16]	1,024

Conv3d-59	[-1, 128, 2, 16, 16]	65,536	ReLU-146	[-1, 512, 2, 16, 16]	0
BatchNorm3d-60	[-1, 128, 2, 16, 16]	256	Conv3d-147	[-1, 2048, 2, 16, 16]	1,048,576
ReLU-61	[-1, 128, 2, 16, 16]	0	BatchNorm3d-148	[-1, 2048, 2, 16, 16]	4,096
Conv3d-62	[-1, 128, 2, 16, 16]	442,368	Conv3d-149	[-1, 2048, 2, 16, 16]	2,097,152
BatchNorm3d-63	[-1, 128, 2, 16, 16]	256	BatchNorm3d-150	[-1, 2048, 2, 16, 16]	4,096
ReLU-64	[-1, 128, 2, 16, 16]	0	ReLU-151	[-1, 2048, 2, 16, 16]	0
Conv3d-65	[-1, 512, 2, 16, 16]	65,536	Bottleneck-152	[-1, 2048, 2, 16, 16]	0
BatchNorm3d-66	[-1, 512, 2, 16, 16]	1,024	Conv3d-153	[-1, 512, 2, 16, 16]	1,048,576
ReLU-67	[-1, 512, 2, 16, 16]	0	BatchNorm3d-154	[-1, 512, 2, 16, 16]	1,024
Bottleneck-68	[-1, 512, 2, 16, 16]	0	ReLU-155	[-1, 512, 2, 16, 16]	0
Conv3d-69	[-1, 128, 2, 16, 16]	65,536	Conv3d-156	[-1, 512, 2, 16, 16]	7,077,888
BatchNorm3d-70	[-1, 128, 2, 16, 16]	256	BatchNorm3d-157	[-1, 512, 2, 16, 16]	1,024
ReLU-71	[-1, 128, 2, 16, 16]	0	ReLU-158	[-1, 512, 2, 16, 16]	0
Conv3d-72	[-1, 128, 2, 16, 16]	442,368	Conv3d-159	[-1, 2048, 2, 16, 16]	1,048,576
BatchNorm3d-73	[-1, 128, 2, 16, 16]	256	BatchNorm3d-160	[-1, 2048, 2, 16, 16]	4,096
ReLU-74	[-1, 128, 2, 16, 16]	0	ReLU-161	[-1, 2048, 2, 16, 16]	0
Conv3d-75	[-1, 512, 2, 16, 16]	65,536	Bottleneck-162	[-1, 2048, 2, 16, 16]	0
BatchNorm3d-76	[-1, 512, 2, 16, 16]	1,024	Conv3d-163	[-1, 512, 2, 16, 16]	1,048,576
ReLU-77	[-1, 512, 2, 16, 16]	0	BatchNorm3d-164	[-1, 512, 2, 16, 16]	1,024
Bottleneck-78	[-1, 512, 2, 16, 16]	0	ReLU-165	[-1, 512, 2, 16, 16]	0

Conv3d-79	[-1, 256, 2, 16, 16]	131,072	Conv3d-166	[-1, 512, 2, 16, 16]	7,077,888
BatchNorm3d-80	[-1, 256, 2, 16, 16]	512	BatchNorm3d-167	[-1, 512, 2, 16, 16]	1,024
ReLU-81	[-1, 256, 2, 16, 16]	0	ReLU-168	[-1, 512, 2, 16, 16]	0
Conv3d-82	[-1, 256, 2, 16, 16]	1,769,472	Conv3d-169	[-1, 2048, 2, 16, 16]	1,048,576
BatchNorm3d-83	[-1, 256, 2, 16, 16]	512	BatchNorm3d-170	[-1, 2048, 2, 16, 16]	4,096
ReLU-84	[-1, 256, 2, 16, 16]	0	ReLU-171	[-1, 2048, 2, 16, 16]	0
Conv3d-85	[-1, 1024, 2, 16, 16]	262,144	Bottleneck-172	[-1, 2048, 2, 16, 16]	0
BatchNorm3d-86	[-1, 1024, 2, 16, 16]	2,048	AdaptiveMaxPool3d-173	[-1, 2048, 1, 1, 1]	0
Conv3d-87	[-1, 1024, 2, 16, 16]	524,288	ResNet-174	[-1, 2048, 1, 1, 1]	0

Supplementary figure1: The prior distributions of scale parameters (Delta) before and after handcrafted radiomic features harmonization

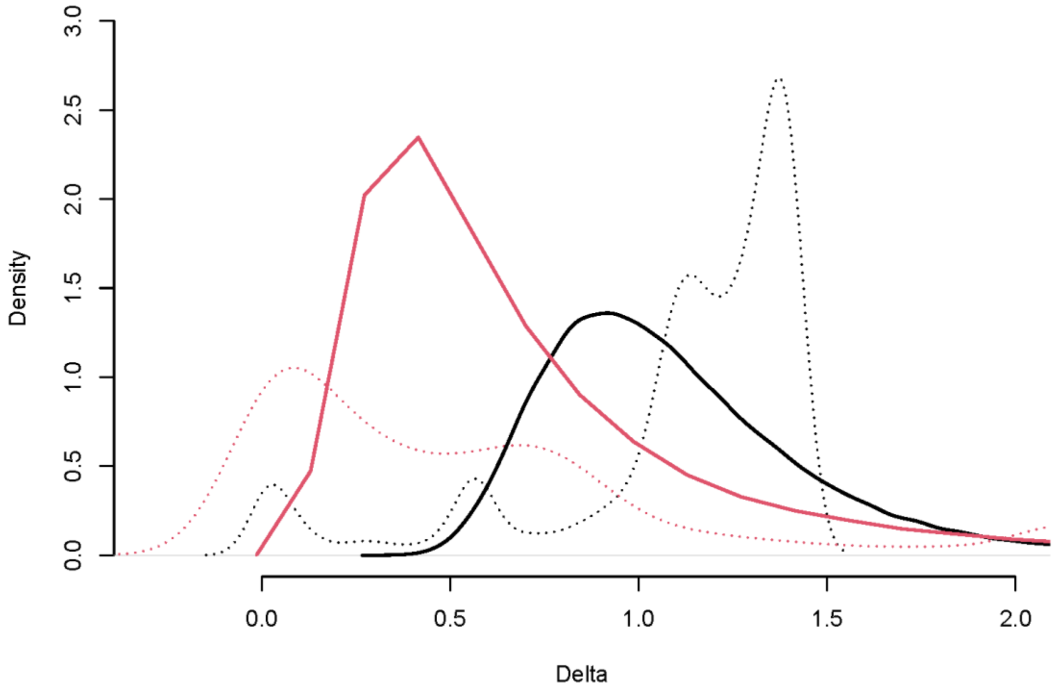

Supplementary figure2: The prior distributions of scale parameters (Gamma) before and after handcrafted radiomic features harmonization.

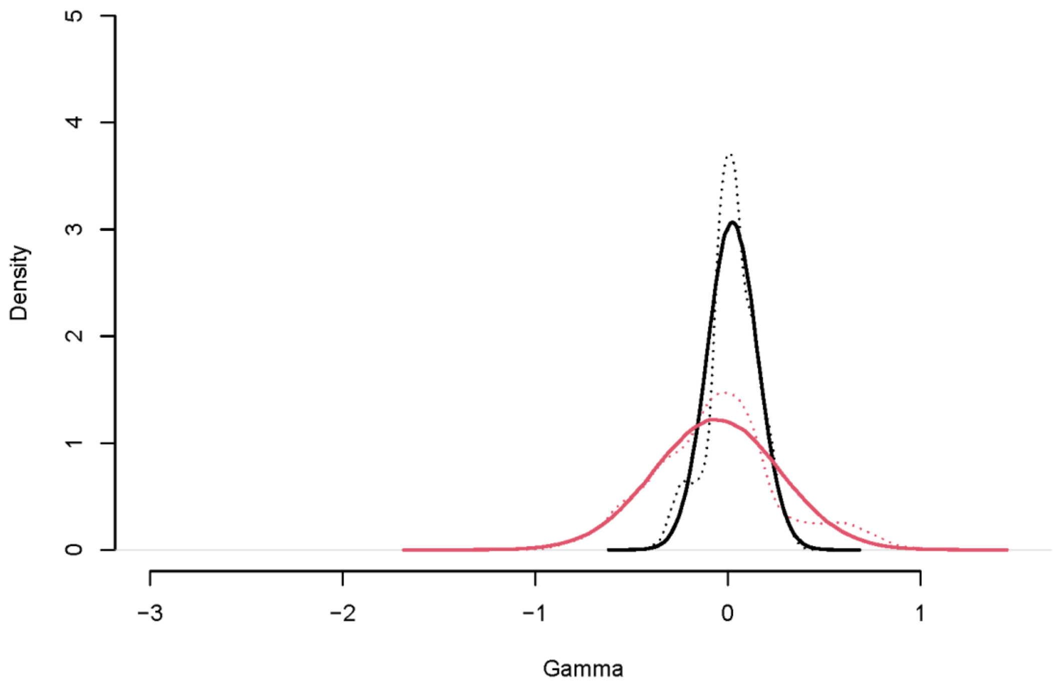

Supplementary figure3: The error bar plot in lasso selection

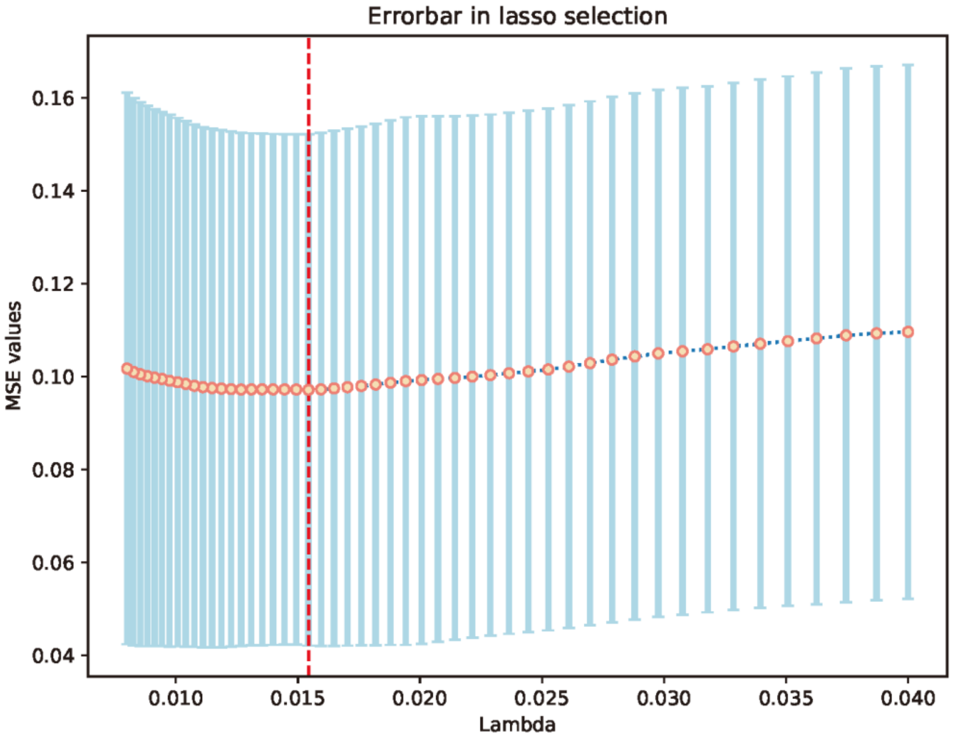

Supplementary figure4 Selected features weights after lasso selection

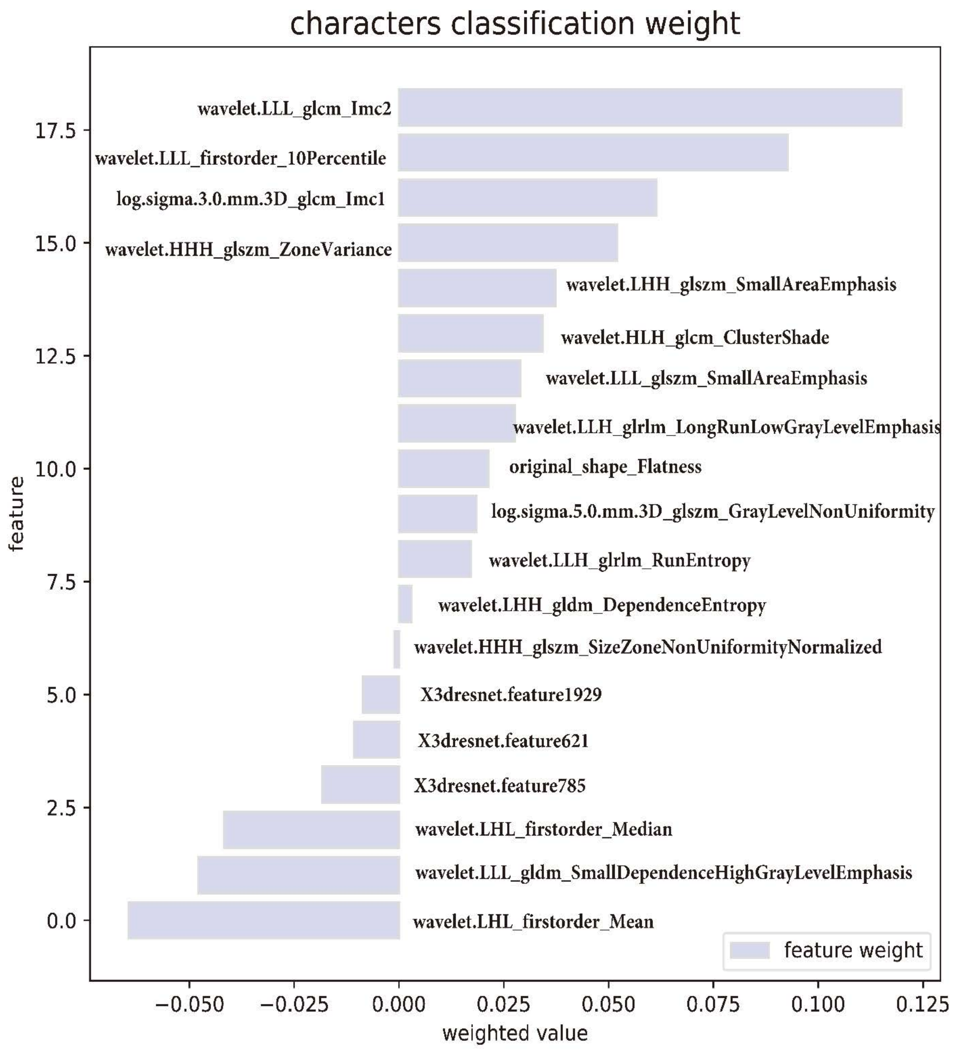

Supplementary figure5: Roc curves for blending voting classifier in external validation dataset.

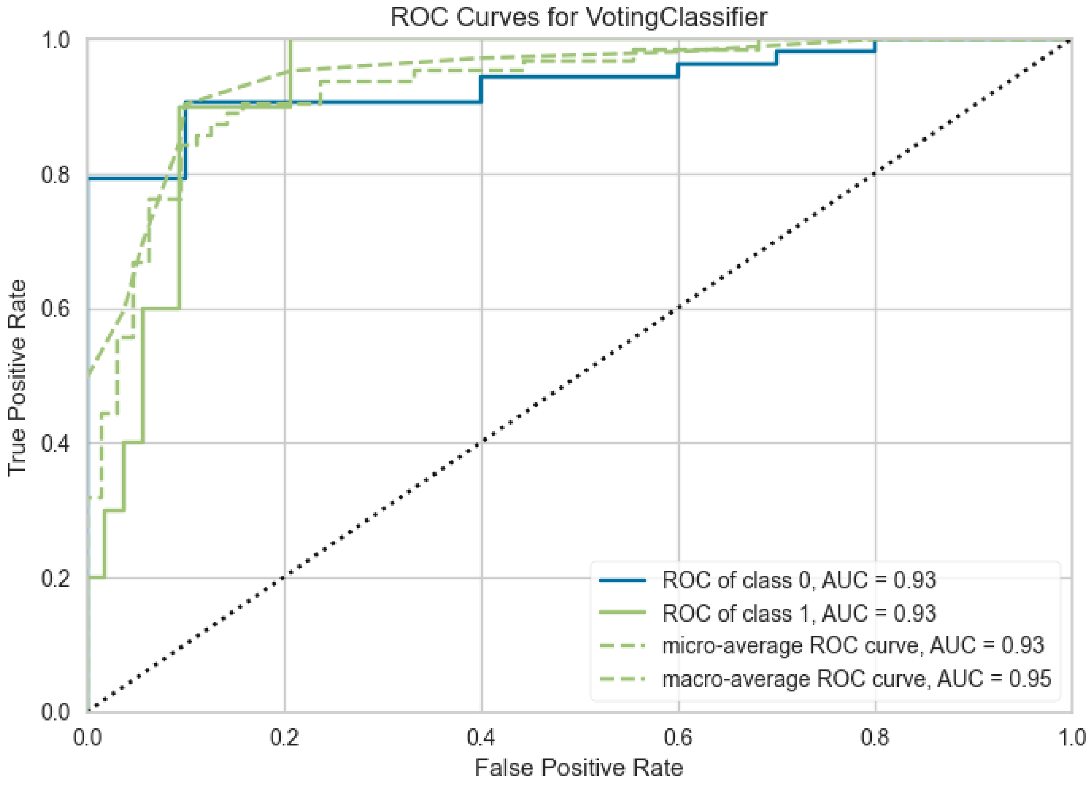

Supplementary figure6: The confusion matrix for the blending model and base models in external validation datasets

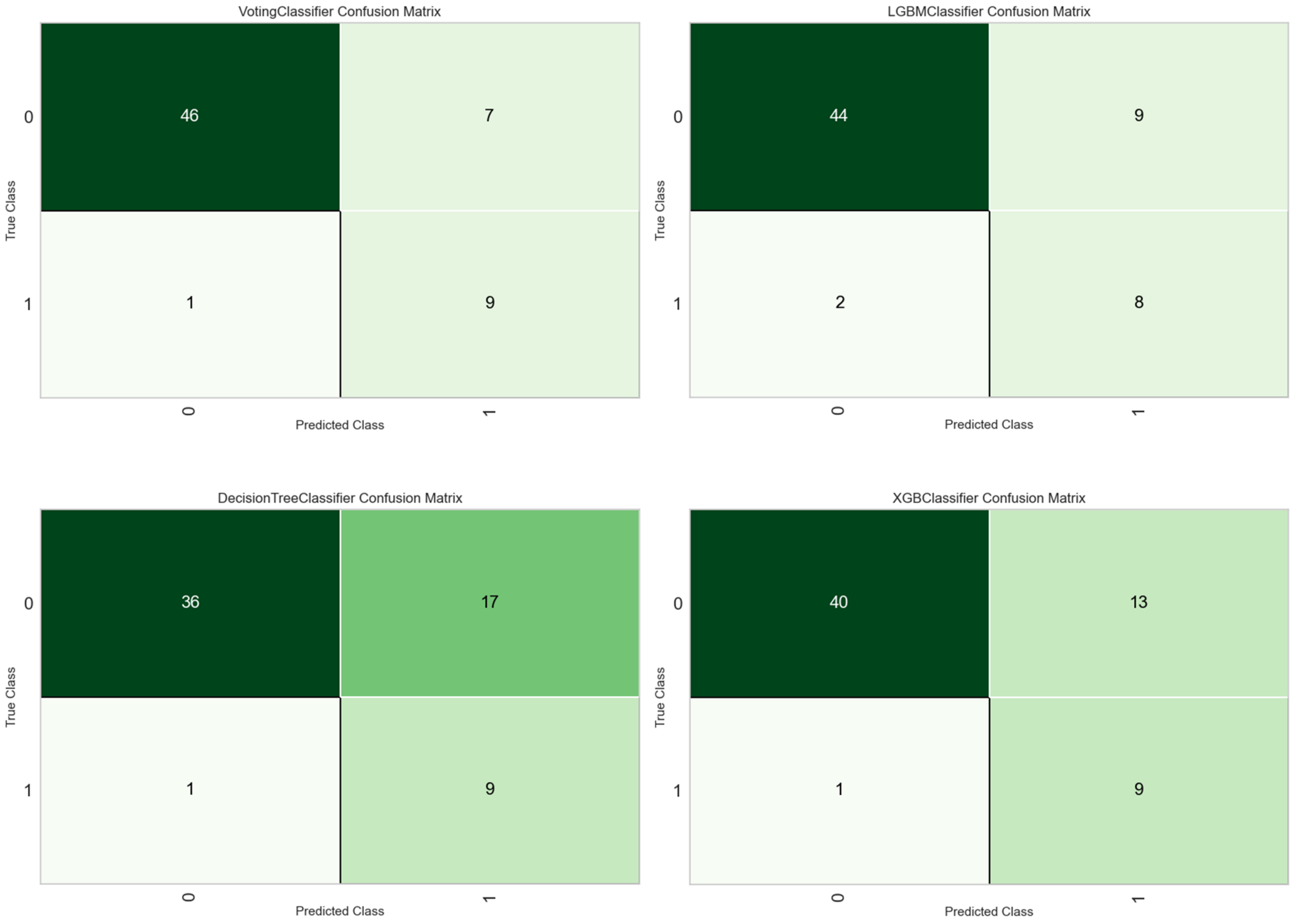

Supplementary figure7: The circle plot shows the correlation between deep learning and handcrafted radiomic features after lasso selection.

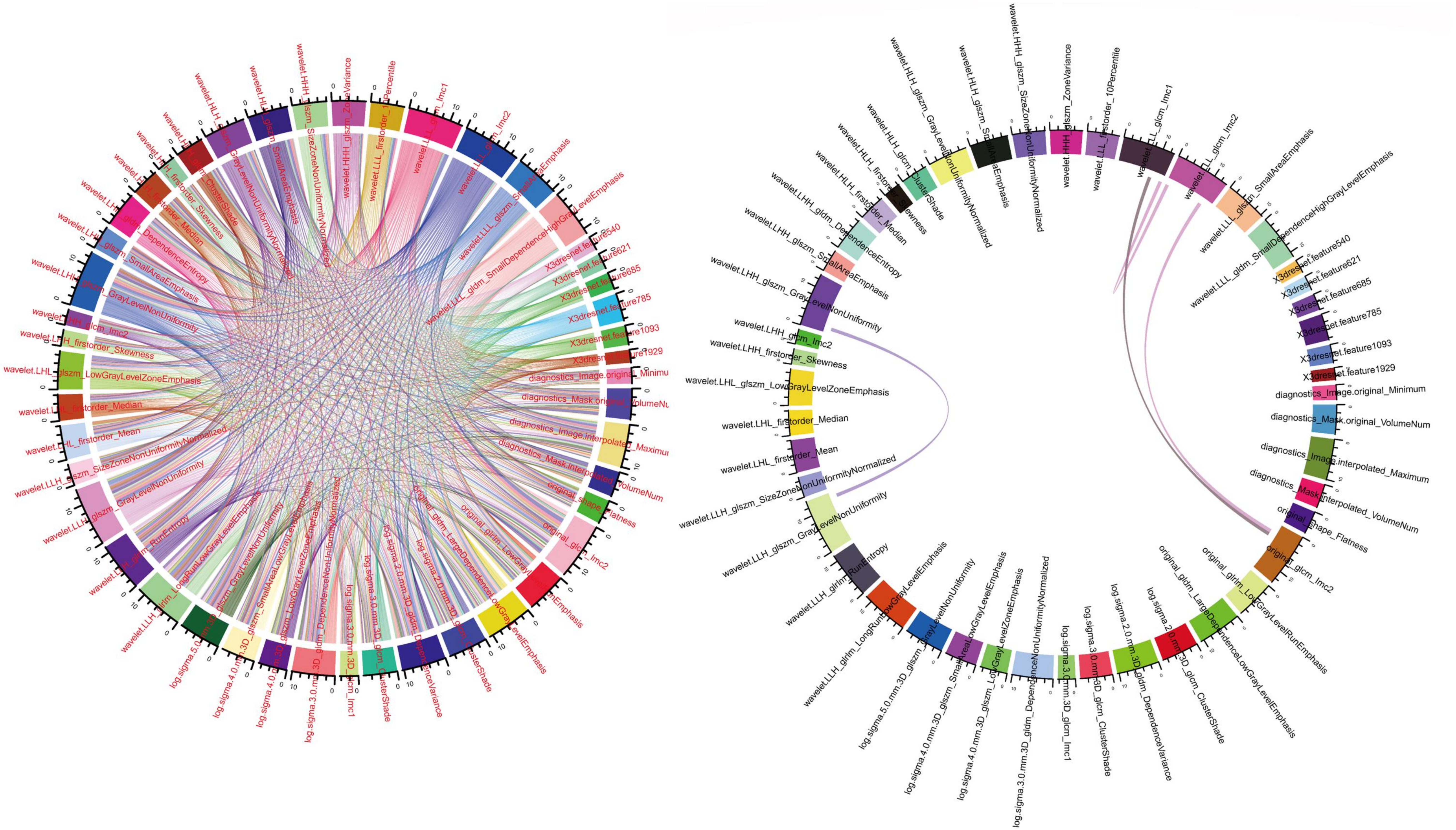

Strong positive correlation: original_glcm_Imc2& wavelet.LLL_glcm_Imc2(0.982), wavelet.LHH_glszm_GrayLevelNonUniformity& wavelet.LLH_glszm_GrayLevelNonUniformity (0.920). Strong negative correlation: wavelet.LLL_glcm_Imc1& original_glcm_Imc2(-0.915), wavelet.LLL_glcm_Imc1& wavelet.LLL_glcm_Imc2(-0.940). The reference value was set to 0.9(absolute value)

Supplementary figure8: Decision boundary plot for four classifiers in external validation dataset.

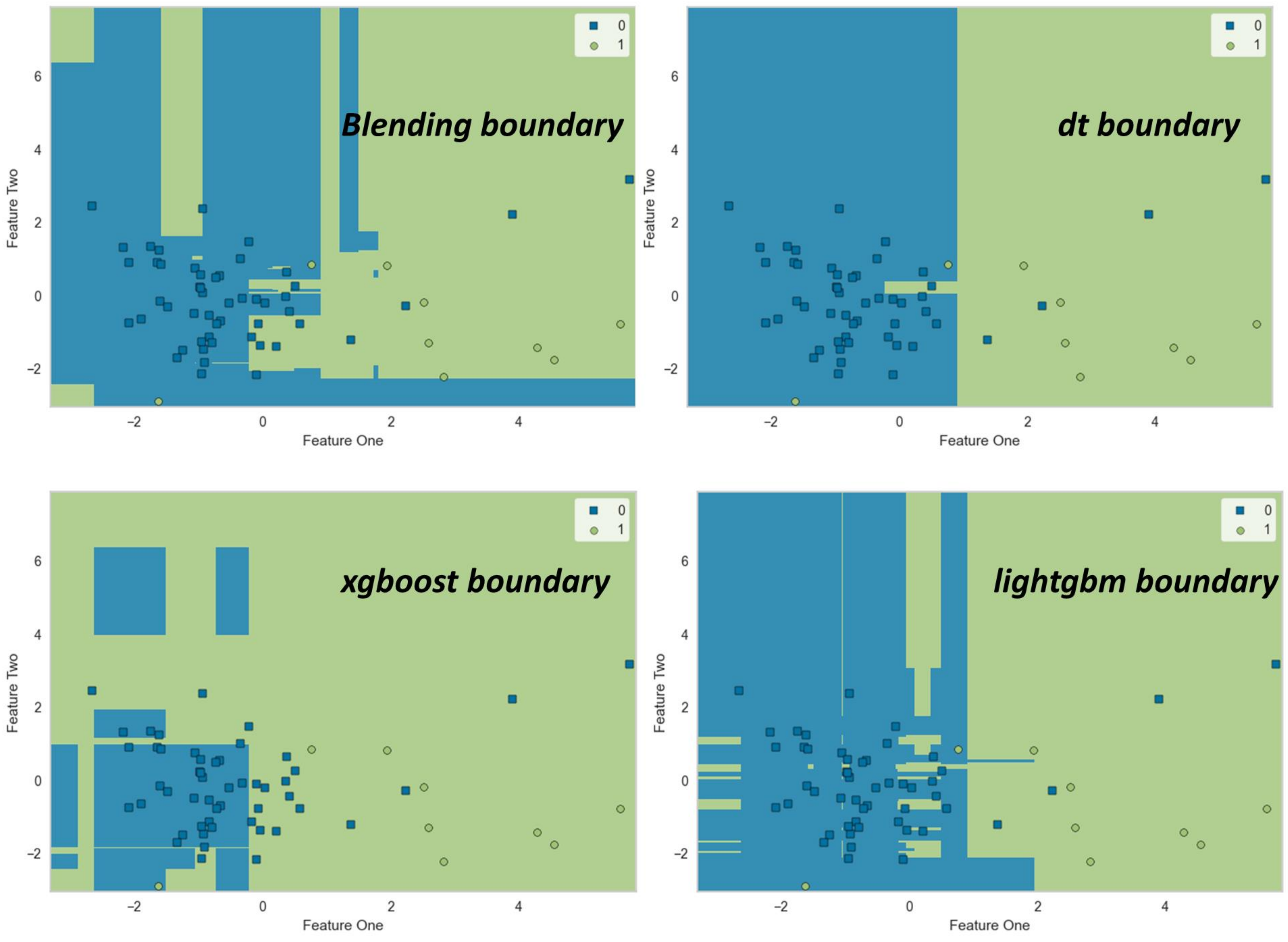

Supplement: Supplementary file 1 — Additional file 1. This material supplements detailed enrollment procedure and quality control methods. [file 13244_2022_1349_MOESM1_ESM.pdf]
